# AQP4 labels a subpopulation of white matter-dependent glial radial cells affected by pediatric hydrocephalus, and its expression increased in glial microvesicles released to the cerebrospinal fluid in obstructive hydrocephalus

**DOI:** 10.1186/s40478-022-01345-4

**Published:** 2022-03-28

**Authors:** Leandro Castañeyra-Ruiz, Ibrahim González-Marrero, Luis G. Hernández-Abad, Emilia M. Carmona-Calero, Marta R. Pardo, Rebeca Baz-Davila, Seunghyun Lee, Michael Muhonen, Ricardo Borges, Agustín Castañeyra-Perdomo

**Affiliations:** 1 CHOC Children’s Research Institute, 1201 W. La Veta Avenue, Orange, CA 92868 USA; 2 Departamento de Ciencias Médicas Basicas, Anatomía, Facultad de Medicina, Universidad de La Laguna, Ofra S/N, 38071 La Laguna, Islas Canarias Spain; 3 Instituto de Investigación y Ciencias de Puerto del Rosario, 35600 Puerto del Rosario, Islas Canarias Spain; 4 Unidad de Farmacologia, Facultad de Medicina, Universidad de La Laguna, Ofra S/N, 38071 La Laguna, Islas Canarias Spain; 5 Neurosurgery Department, CHOC Children’s Hospital, 505 S Main St., Orange, CA 92868 USA

**Keywords:** AQP4, Hydrocephalus, Gliogenesis, Glial stem cells, Reactive astrogliosis, Microvesicles

## Abstract

Hydrocephalus is a distension of the ventricular system associated with ventricular zone disruption, reactive astrogliosis, periventricular white matter ischemia, axonal impairment, and corpus callosum alterations. The condition's etiology is typically attributed to a malfunction in classical cerebrospinal fluid (CSF) bulk flow; however, this approach does not consider the unique physiology of CSF in fetal and perinatal patients. The parenchymal fluid contributes to the glymphatic system, and plays a fundamental role in pediatric hydrocephalus, with aquaporin 4 (AQP4) as the primary facilitator of these fluid movements. Despite the importance of AQP4 in the pathophysiology of hydrocephalus, it’s expression in human fetal life is not well-studied. This manuscript systematically defines the brain expression of AQP4 in human brain development under control (n = 13) and hydrocephalic conditions (n = 3). Brains from 8 postconceptional weeks (PCW) onward and perinatal CSF from control (n = 2), obstructive (n = 6) and communicating (n = 6) hydrocephalic samples were analyzed through immunohistochemistry, immunofluorescence, western blot, and flow cytometry. Our results indicate that AQP4 expression is observed first in the archicortex, followed by the ganglionic eminences and then the neocortex. In the neocortex, it is initially at the perisylvian regions, and lastly at the occipital and prefrontal zones. Characteristic astrocyte end-feet labeling surrounding the vascular system was not established until 25 PCW. We also found AQP4 expression in a subpopulation of glial radial cells with processes that do not progress radially but, rather, curve following white matter tracts (corpus callosum and fornix), which were considered as glial stem cells (GSC). Under hydrocephalic conditions, GSC adjacent to characteristic ventricular zone disruption showed signs of early differentiation into astrocytes which may affect normal gliogenesis and contribute to the white matter dysgenesis. Finally, we found that AQP4 is expressed in the microvesicle fraction (*p* < 0.01) of CSF from patients with obstructive hydrocephalus. These findings suggest the potential use of AQP4 as a diagnostic and prognostic marker of pediatric hydrocephalus and as gliogenesis biomarker.

**Supplementary Information:**

The online version contains supplementary material available at 10.1186/s40478-022-01345-4.

## Introduction

Hydrocephalus is an active distension of the brain's ventricular system, caused by inadequate passage of cerebrospinal fluid (CSF) from its production points within the ventricular system to its points of absorption into the systemic circulation [57]. In general, the neuropathology of hydrocephalus involves neuroinflammation, alterations in the periventricular white matter, parenchymal stretch associated with compression, and axonal loss. It is also related to neurodevelopmental alterations such as ventricular zone disruption and impairments in neuronal differentiation, including glial radial cell (GRC) loss or dendritic and synaptic atrophy [20, 26, 34, 35, 61]. All these phenomena are accompanied by reactive astrogliosis [29, 44]. Hydrocephalus is classified as obstructive when there is a blockage that impedes the passage of CSF from the ventricular system and as communicating hydrocephalus when the CSF can flow from the ventricles to the subarachnoid space, where absorption is impeded [17]. The obstructive or communicating nature of this pathology is relevant to deciding appropriate treatments since obstructive hydrocephalus is often treated through ventriculostomy of the third ventricle [50]. Pediatric hydrocephalus affects approximately 1 in 1100 children in the United States, and its primary treatment is a diversion of the CSF [33].

The treatment of pediatric hydrocephalus is based on a traditional understanding of CSF flow known as bulk flow theory [18, 19]. The CSF is produced inside the ventricles by the choroid plexus (ChP), and to a lesser extent, ependymal cells, and then flows through the ventricular system, exiting toward the subarachnoid space through the foramina of Lushka and Magendie. Ultimately, the CSF is absorbed through the Pacchionian bodies at the superior sagittal sinus. This approach to the treatment of hydrocephalus has been challenged in the context of pediatric patients since it has been suggested alternative absorption pathways in fetal and perinatal life [52]. Thus, new CSF flow pathways in the brain parenchyma involving deep vascular structures and lymphatic channels have recently been reported [51]. These pathways seem to play a fundamental role in CSF reabsorption during perinatal and infantile life and are associated with the development of pediatric hydrocephalus [51]. The "glymphatic system" was coined to describe this parenchymal CSF pathway because it involves astrocytes and perivascular channels that resemble the lymphatic system [30–32]. The glymphatic system is a flow clearance pathway through which subarachnoid CSF is driven along with the vascular system, flowing into the brain parenchyma through the perivascular space of penetrating arteries and capillaries. CSF is exchanged with interstitial fluid, which ultimately is drained through the perivenous space. This perivascular space, also known as Virchow-Robin space, is surrounded by astrocyte end-feet, which express polarized AQP4 to facilitate bidirectional permeability between the parenchymal interstitium and the Virchow-Robin space [30–32]. AQP4 is also expressed in the basolateral membranes of ependymal cells [2, 49, 55]. In animal models, the expression pattern exclusively associated with astrocyte end-feet and the basolateral membrane of ependymal cells is not achieved until postnatal ages [63]. A limited number of studies have investigated the expression of AQP4 during the fetal stage of human development [5, 28], reporting complex patterns of expression. Experiments with AQP4 knockout mice have suggested that this water channel plays a significant role in maintaining the integrity of the ventricular zone and in maintaining homeostasis between ventricular fluid and brain fluid [22, 40, 66].

The etiology of pediatric hydrocephalus has been associated with disrupted integrity of the ventricular zone [15, 16, 29, 36, 44]. Interestingly, the expression of aquaporin water channels (AQPs) has been detected in the CSF in patients with inflammatory conditions such as meningitis or hydrocephalus; however, how these water channels are released into the CSF is unknown [7, 11, 12, 14, 41]. In recent decades, researchers have defined a new mechanism of cellular communication based on the intercellular transfer of extracellular vesicles. These vesicles serve as vehicles to transport cytosolic proteins, lipids, and RNA [54]. We, therefore, wondered whether the expression of AQP4 in CSF was associated with microvesicle release. Since AQP4 plays a fundamental role in parenchymal CSF flow [38, 45, 62], this study aimed to characterize the expression of AQP4 during fetal and perinatal life in human tissue and CSF microvesicles samples under hydrocephalic and control conditions to elucidate the role of this water channel in brain development and pediatric hydrocephalus.

## Materials and methods

### Brain tissue

Fourteen fetal brains, one newborn brain, and one adult brain were obtained from the University Hospital of Canary Islands, in compliance with the legal requirements of Law 42/1988, of December 28 (Official bulletin of Spain, 12/31/1988). In this study, fetus age was determined from the gestational history reported by the obstetrician. Pathological characteristics were determined by the Pathological Anatomy Service of the University Hospital of Canary Islands (Table 1). Fetal brains were obtained after legal abortions following national guidelines in Spain either with not known neurological pathologies or with pathologies that present ventriculomegaly such as Arnold Chiari II, lissencephaly, or myelomeningocele. No other pathologies were attributed to the patients. The adult brain was from a 34-year-old male patient with pancreatic cancer and no neurological symptoms. The perinatal brain was from a child that died shortly after birth due to respiratory failure. The protocol for the use of human samples in the present study was approved by the Ethics Committee of the University of La Laguna, according to the 2004 Helsinki Declaration. Brains were fixed with 10% formalin, followed by post-fixation in Bouin or Carnoy solution. The tissue was then immersed for 24 h in a series of 70, 80, 90, 96, and 100% alcohol solutions. Once dehydrated, samples were exposed to methyl benzoate and 100% alcohol in equal parts for 24 h, 100% methyl benzoate for 24 h, a mixture of equal parts benzol and paraffin for 30 min at 60 ºC, and paraffin for 24 h at 60ºC. Finally, we let the paraffin dry at room temperature to form the paraffin blocks with the tissue embedded. The blocks were sectioned with a microtome (Minot, 1212, Leitz Wetzlar, Germany) into serial sections of 10-μm thickness to obtain slices of the areas of interest. Allen atlas was used for neuroanatomical confirmation [37].

**Table 1:** Brain samples used for the study

Control brains		Brains with hydrocephalus		
Sample	PCW	Sample	PCW	Pathology
1	8	14	21	Lissencephaly
2	9	15	21	Myelomeningocele
3	12	16	25	Arnold Chiari II
4	13	–	–	–
5	15	–	–	–
6	15–16	–	–	–
7	17	–	–	–
8	20	–	–	–
9	21	–	–	–
10	25	–	–	–
11	27	–	–	–
12	40	–	–	–
13	34 years	–	–	–

*PCW* post-conceptional weeks

### Cerebrospinal fluid

CSF samples were obtained through the pediatric surgery service and the microbiology service at the University Hospital of Canary Islands, in cases where CSF had been removed for diagnostic or therapeutic reasons. Hydrocephalic CSF samples were extracted from the lateral ventricle during the first five days of life to lower the intracranial pressure. Control samples were obtained by lumbar puncture in patients with suspected meningitis or encephalitis (Table 2). Informed consent to participate in the study was obtained from the parents of all patients included in the study, and the medical ethics committee of the University Hospital of Canary Islands approved the study. Ultrasound was used to diagnose hydrocephalus during pregnancy. The cases with tetraventricular ventriculomegaly with no obstruction were considered communicating hydrocephalus. On the other hand, the cases that showed obstruction either in the mesencephalic aqueduct (mesencephalic aqueduct obstruction) or in the fourth ventricle outlet due to a lack of patency of the foramina of Lushka and Megendie (Dandy-Walker syndrome), or blood clots (post-hemorrhagic hydrocephalus) were considered obstructive hydrocephalus.

**Table 2:** CSF samples used for the study

CSF	Pathology	Age
1	MAO	1–5 days
2	MAO	1–5 days
3	MAO	27 PCW
4	MAO	1 year
5	MAO	1–5 days
6	DW	1–5 days
7	PHH	1–5 days
8	PHH	1–5 days
9	4 V	1–5 days
10	4 V	1–5 days
11	4 V	38 PCW
12	4 V	1–5 days
13	Cont	3 days
14	Cont	38 PCW

*4V* tetraventricular hydrocephalus, *Cont* control, *DW* Dandy Walker malformation, *MAO* mesencephalic aqueduct obstruction, *PHH* post-hemorrhagic hydrocephalus

### Immunohistochemistry

Sections were deparaffinized by immersion in xylol for 30 min. Subsequently, sections were hydrated through an alcohol series of decreasing gradation (100, 96, 90, 80, and 70% ethanol). Sections were immersed in phosphate buffered saline (PBS) at pH 7.4 Na_2_HPO_4_·2H_2_O 1.9%, KH_2_PO4 0.43%, NaCl 7.22%) and boiled in sodium citrate buffer (pH 6) for 20 min to unmask the epitopes. Primary antibodies (Table 3) were incubated overnight in a humidity chamber at room temperature. Secondary antibodies were incubated for one hour (Table 3). Sections were treated with H_2_O_2_ 0.3% for 20 min to avoid unspecific endogenous peroxidase labeling. DAB (3,3-diaminobenzidine C12H14N4, Sigma-Aldrich) 0.03% and H_2_O_2_ 0.003% plus ammonium nickel sulfate (H_8_N_2_NiO_8_S_2_·6H_2_O) 0.04% were used to visualize the immunoreaction. Finally, slides were dehydrated in increasing concentrations of alcohols (70, 80, 96, and 100%), passed through xylol, and mounted with Eukitt mounting media. Sections were visualized with a LEICA DMRB microscope with a LEICA DC 300F CCD camera.

**Table 3:** Antibodies used for immunohistochemistry (IHC) and western blot (WB)

Antibody	Host	Dilution IHC	Dilution WB	Dilution Flow cytometry	Lot number, manufacturer
GFAP	Rabbit	1:400	1:1000	–	#7260, Abcam
GFAP	Rat	1:200	–	1:100	#ab279291, Abcam
AQP4	Rabbit	1:1500	1:1000	1:100	#PAB 20767, Abnova
S100ß	Rabbit	1:100	–	–	#Ab52642, Abcam
Transthyretin	Rabbit	–	1:1000	–	#A0002, Dako
Vimentin	Rabbit	1:200	–	–	#ab92547, Abcam
Vimentin	Mouse	1:200	–	–	#ab8978, Abcam
Anti-mouse Alexa Fluor 555	Goat	1:300	–	–	#AQ21422, Thermo Fisher Scientific
Anti-rabbit Alexa Fluor 488	Goat	1:300	–	–	#A11034, Thermo Fisher Scientific
Anti-rat FITC	Goat	–	–	1:100	#Ab6840, Abcam
Anti-rabbit APC	Goat	–	–	1:100	#Ab130805, Abcam
Peroxidase-conjugated anti-rabbit		–	1:10,000	–	#70742, Cell Signaling Technology

### Immunofluorescence

After deparaffinization and hydration, sections were incubated with the first antibody overnight (~ 15 h). Sections were then washed in PBS (pH 7.3) and incubated for one hour with the corresponding secondary antibody (Invitrogen) at 1:300 dilution, conjugated to a fluorophore, Alexa Fluor 488 (green) or 555 red (Invitrogen) (Table 3). Samples were incubated for 5 min with DAPI (1: 5000, mounted in equal parts PBS + glycerol), and viewed on an Olympus Fluoview 1000 Spectral Confocal Microscope.

### CSF Vesicle Extraction

CSF was initially stored at − 80 °C, and then thawed on ice, at which point 1 mL of the sample was introduced into a 2-mL Eppendorf tube, and 1 mL PBS + Complete was added at 2X. Samples were then sequentially centrifuged, 10 min at 2500*g* to discriminate cells from cell debris, yielding pellet 1 (P1) and supernatant (S1). S1 was centrifuged at 17,000*g* for 15 min to yield pellet 2 (P2) and supernatant 2 (S2). S2 was introduced into a 5-mL polypropylene tube (polypropylene, Thinwall, 5.0 mL, 13 × 51 mm Beckman Coulter), and 3.8 mL of 1X PBS + Complete was added; later, samples were ultracentrifuged in a Beckman Coulter Optima L-90 K ultracentrifuge with an oscillating SW55 Ti rotor, for 70 min at 200,000*g*, yielding pellet 3 (P3) and supernatant 3 (S3). This differential centrifugation method allowed the fractional isolation of different vesicle pools, including exosomes, and has been modified from Bianco et al. [4]. Finally, pellets were resuspended in SDS buffer to perform western blot or resuspended in 100 µl of PBS with 0.01% formalin to fix the vesicles for flow cytometry analysis. Total protein was precipitated from S3 using acetone. Four parts acetone (− 20 °C) were added to the sample, which was shaken vigorously and left for 30 min at -20ºC, then centrifuged at 15,000×*g* for 10 min and resuspended in SDS buffer for western blotting (centrifugation was performed at 4 °C).

### Western blot analysis

For western blot analysis, CSF was included in the sample buffer (100 mM Tris–HCl pH 6.8, 4% SDS, 2% bromophenol blue, 20% glycerol, 0.5% β-mercaptoethanol) and heated at 95 °C for 5 min to denature the proteins. Electrophoresis was performed in 10% polyacrylamide gels at 35 mA. Proteins were transferred to a PVDF membrane (Millipore) at 400 mA for 90 min in transfer buffer (39 mM glycine, 48 mM Tris-base pH 8.3, 0.037% SDS, 10% methanol) in a Mini-Trans Blot (Bio-Rad) system. After 1 h of blocking with 5% dehydrated milk in tris-saline buffer (TBS), membranes were incubated overnight with primary antibodies (Table 3) at 4 °C. Protein concentration was detected with chemiluminescence (Amersham Biosciences) after one-hour incubation with horseradish peroxidase-conjugated anti-rabbit antibodies (Jackson Immuno Research Laboratories) (Table 3). Chemiluminescence was detected with ChemiDoc MP, (Biorad) and analyzed with ImageLab software.

### Flow cytometry

Flow cytometry was used to quantify the expression of AQP4 and GFAP in individual microvesicles. Extracted vesicles were incubated with primary antibody for one hour (Table 3); then washed with 1 mL PBS. Samples were centrifuged and resuspended in 100 µL PBS and incubated with secondary antibodies (Table 3). For each sample, 1600 events (vesicles) were analyzed on a MacsQuant Analyzer 10 flow cytometer (Miltenyi Biotec, Germany), with data represented on a logarithmic scale. Data analysis was performed with the MacsQuantify program. Mann–Whitney U test was used only to compare obstructive (n = 6) and communicating (n = 6) groups. Results were considered statistically significant if *p* < 0.05. Data are presented as group medians using Prism 5 (GraphPad Software, San Diego, CA).

Fluorescence from secondary antibodies was considered as background. A confocal spectral microscope (Olympus Fluoview 1000) was used to confirm the presence of microvesicles (Additional file 1: Fig. S2).

## Results

### AQP4 expression in developing human brain

**Fig. 1:**
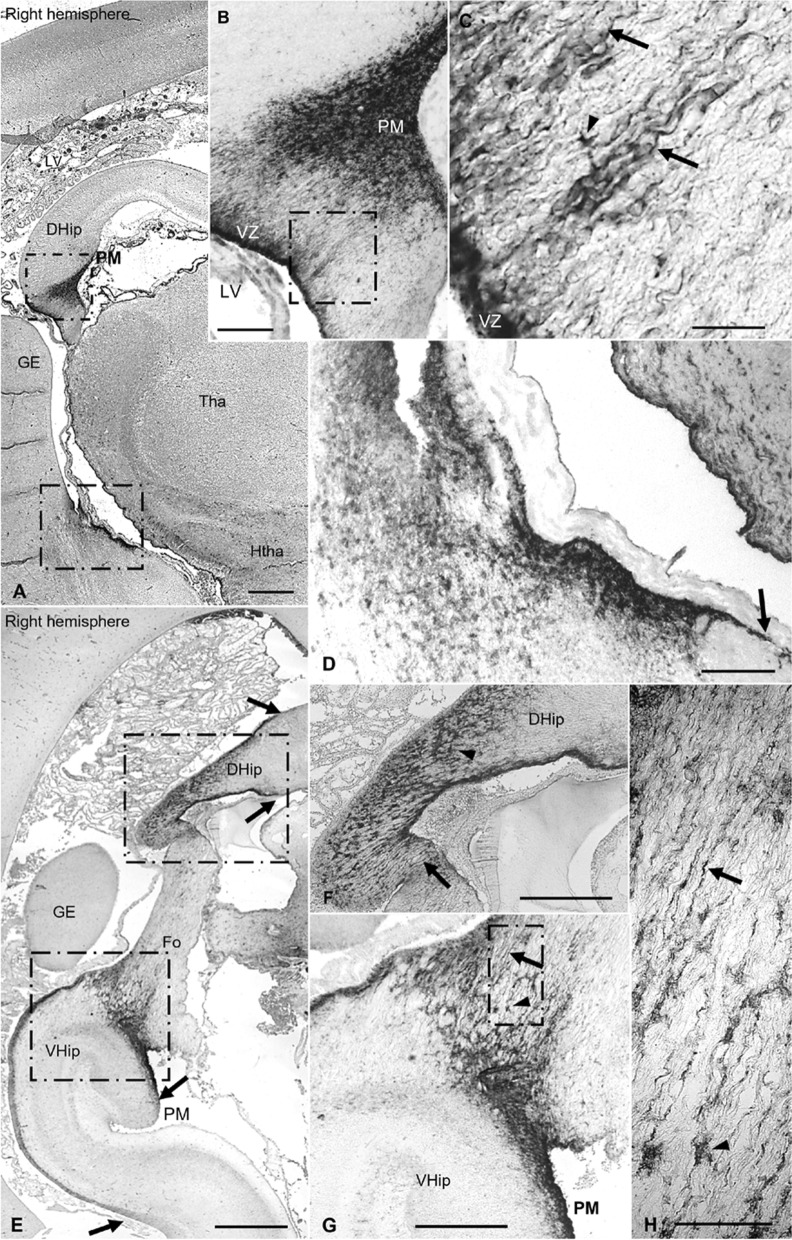
AQP4 immunoreactivity in the archicortex of control samples at 12 and 17 PCW **a** Panoramic image of a coronal brain section, 13 PCW. **b** Magnification of the square in A, indicating selective expression of AQP4 in dorsal hippocampus, mostly in the VZ and PM zones. **c** Magnification of the square in **b**, indicating that AQP4 is expressed in radial processes similar to those associated with radial glial cells (arrows) and in astrocyte cellular bodies (arrowhead). **d** Magnification of the area where the ventral hippocampus will develop. Arrow indicates that immunoreactivity is restricted to the VZ associated with the future archicortex. **e** Panoramic image of a coronal section of fetal brain at 17 PCW. Arrows indicate that expression is still associated with VZ and PM zones in the archicortex but has also expanded dorsally through degenerating dorsal hippocampus and ventrally toward the definitive hippocampus. **f** Magnification of the square on the dorsal hippocampus in E. Arrowhead indicates AQP4 expression in cellular bodies of degenerating dorsal hippocampus and arrow indicates radial processes. **g, h** Magnification of ventral hippocampus. Arrowheads indicate immunoreactivity of cell bodies. Arrows indicate expression in glial radial cell-like cells, with processes that progress toward the fornix, not radially. DHip, dorsal hippocampus; Fo, fornix; GE, ganglionic eminence; Htha, hypothalamus; LV, lateral ventricle; PCW, post-conceptional weeks; PM, pia mater; Tha, thalamus; VHip, ventral hippocampus; VZ, ventricular zone. Scale bars: **a** 500 µm; **b** 250 µm; **c** 30 µm; **d** 60 µm; **e** 600 µm; **f, g** 300 µm; **h** 75

The earliest observed APQ4 expression was detected at 13 PCW in the glioepithelium of dorsal hippocampus (Fig. 1a–c). No expression was detected at 8 or 10 PCW (Additional file 1: Fig. S1). Labeling was stronger on the pial surface than in the ventricular zone (VZ). Interestingly, we found AQP4-positive cells with a basal process projected toward the pia mater (PM). Thus, these cells connect the VZ with the PM as glial radial cells (GRC) do. At 15 PCW, there was no discernible change in the patter of AQP4 expression, except that AQP4 was expressed on GRCs progressing toward incipient fornix (Fo) (Additional file 1: Figure S1). At 17 PCW, degeneration of the dorsal hippocampus (DHip) was observed, in which the gray matter fades out with replacement by AQP4-positive nerve projections that would form part of the CC. At this point, AQP4 expression was observed throughout the degenerating DHip, with staining expanding dorsally and ventrally along the VZ of the medial wall of the lateral ventricle in the archicortex (Fig. 1e). The ventral hippocampus (VHip) showed immunoreaction in the apical and basal surfaces of the glioepithelium. We also found AQP4-positive GRC-like cells following the fornical fibers longitudinally (Fig. 1g, h).

**Fig. 2:**
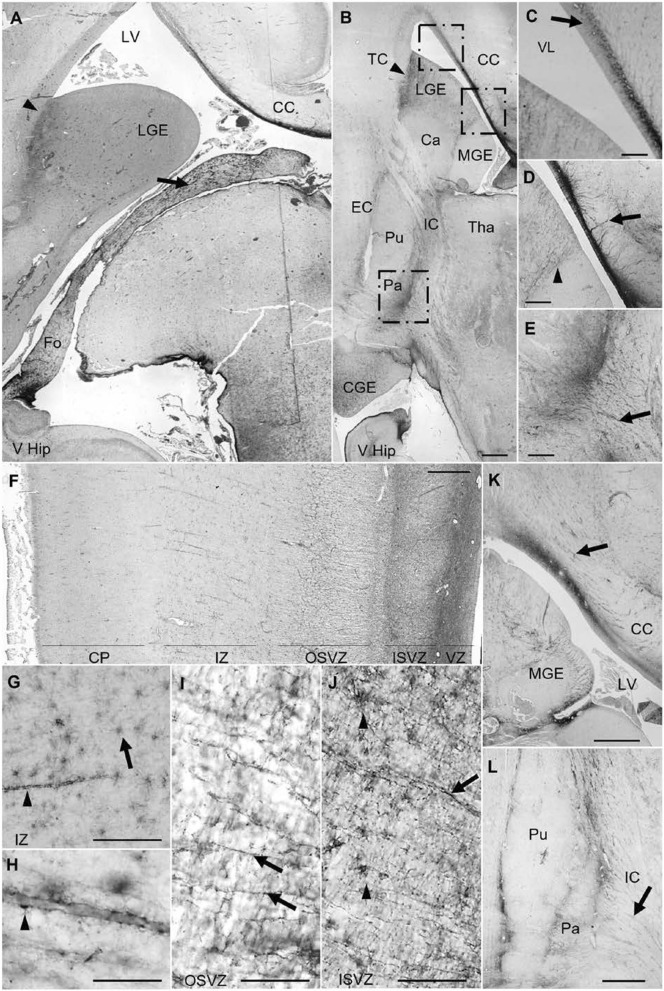
AQP4 immunoreactivity in control samples at 20, 21 and 25 PCW. **a** Panoramic view of a coronal section from a 20 PCW brain. AQP4 expression has expanded to the entire fornix (arrow) and the lateral ganglionic eminence (arrowhead). **b** Panoramic view of a coronal slice of 21 PCW. Arrowhead indicates boundary between lateral ganglionic eminence and cortex. **c** Magnification of the square in B. Arrow indicates specific staining of the subventricular zone of the CC, with decreasing intensity from dorsomedial to dorsolateral. **d** Magnification of the corpus callosum and basal ganglia. Arrow indicates penetration of the corpus callosum by cellular processes. Arrowhead indicates AQP4 expression at the limit between the lateral and medial ganglionic eminence. **e** Magnification of the ventromedial zone of the globus pallidum and the internal capsule. Arrow indicates a cellular process penetrating into the capsular fibers. **f** Coronal section image of the lateral parieto-occipital neocortex at 25 PCW. **g, h** Magnification of the intermediate zone showing AQP4 immunoreactivity in astrocyte bodies (arrow) and astrocyte end-feet surrounding the blood vessels forming the neurovascular unit (arrowheads). **i, j** Detail of the outer and inner subventricular zone of the lateral parieto-occipital neocortex. Arrows indicate immunoreactive radial processes and arrowheads indicate cellular bodies. **k** Coronal section image at a central level showing ganglionic eminences and corpus callosum. Arrows indicate immunoreactive processes that do no progress radially but follow the corpus callosum fibers in the same plane as the section. **l** Image of the lenticular nucleus (putamen and globus pallidum). Arrows indicate cellular processes extending toward the internal capsule. Ca, caudate; CC, corpus callosum; CGE, caudal ganglionic eminence; CP, cortical plate; EC, external capsule; Fo, fornix; IC, internal capsule; ISVZ, inner subventricular zone; IZ, intermediate zone (subplate); LGE, lateral ganglionic eminence; LV, lateral ventricle; MGE, medial ganglionic eminence; OSVZ, outer subventricular zone; Pa, pallidum; PM, pia mater; Pu, putamen; Tha, thalamus; IC, Isocortex; VHip, ventral hippocampus; VZ, ventricular zone. Scale bars: **a** 250; **b** 250 µm; **c–e** 90 µm; **f** 320 µm; **g, i, j** 240 µm; **h** 80 µm; **k, l** 500 µm

At 20–21 gestational weeks, AQP4 expression had reached the entire Fo (Fig. 2a). Immunoreactivity was observed in the VZ of the CC, progressing dorsolaterally toward the roof of the lateral ventricle and through the subventricular zone (SVZ) (Fig. 2a–c). AQP4 was expressed in GRC processes that penetrated the fibers of the CC without crossing it (Fig. 2c, d). During this gestational period, AQP4 was selectively expressed in the lateral and caudal ganglionic eminences (LGE, CGE), defining an anatomical boundary between these eminences and the telencephalic structures that surround them; medial ganglionic eminence (MGE), Isocortex (IC) and caudate (Ca), which were negative for AQP4 expression. The putamen (Pu) was also immunoreactive in the areas adjacent to the external capsule (EC). the globus pallidus (Pa) was markedly defined, with expression in cellular processes projected towards capsular nerve fibers (Fig. 2b, d, e).

At 25 gestational weeks, the MGE was immunoreactive, and continued immunoreactivity was observed in cell processes at the CC and the internal capsule (IC). During this gestational period, AQP4 expression was not only associated with the main nervous tracts but also detectable for the first time throughout the neocortex, marking radial processes in the inner and outer SVZ as well as astrocytes in the intermediate zone (IZ) (Fig. 2f-j). It is in this area where AQP4 was first expressed in the astrocyte end-feet surrounding the blood vessels to form the neurovascular unit (Fig. 2f, g).

In adulthood (34 years), AQP4 immunoreactivity is primarily detected in the astrocytes end-feet of white matter, with diffuse immunoreactivity in a dot-like pattern. It is also expressed surrounding the blood vessels supplying gray matter (Additional file 1: Fig. S1).

### AQP4, a marker of glial lineage

#### AQP4 and vimentin

**Fig. 3:**
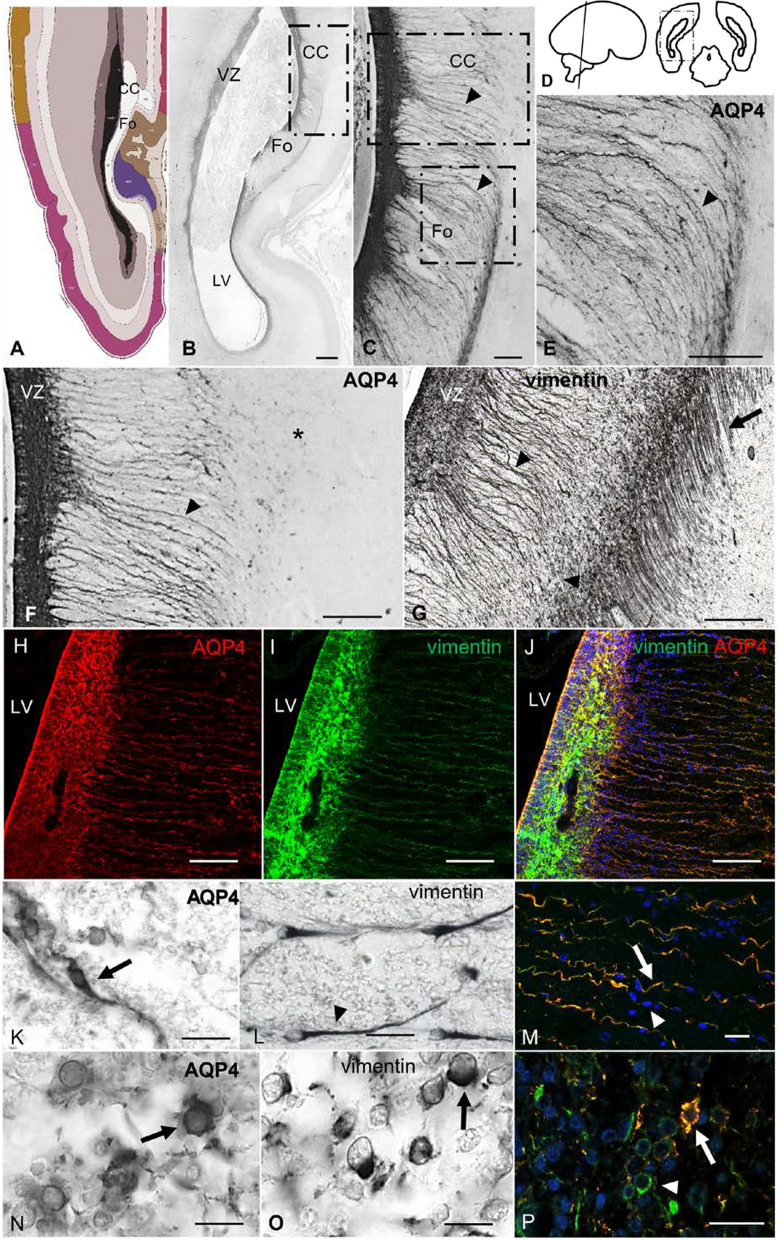
AQP4 and vimentin immunoreactivity in a control sample of 21 PCW. **a** Schematic representation (modified from Allen atlas) at 21 PCW showing localization of the corpus callosum and the fornix. **b** Panoramic view from a parietooccipital section of a 21 PCW brain. AQP4 expression is restricted to VZ and the main white matter tracts (corpus callosum and fornix). **c** Magnification of the square in B showing staining for glial radial cell-like cells associated with white matter tracts (arrowheads). **d** Schematic representation of the area of study. **e** Magnification of the square in **c**, showing basal processes that curve to follow the fiber tracts of the fornix (arrowhead) and do not progress radially toward the cortical plate. **f** Magnification of the square in **c** showing expression in VZ and radial glial cell-like cells (arrowhead) specific to the corpus callosum with progression toward the cortical plate (asterisk). G; Vimentin expression in a parallel slide from F showing immunoreactivity in the VZ, cell processes of the corpus callosum (arrowhead), and glial radial cell processes that project to the cortical plate (arrow). **h–j** Immunofluorescence for AQP4 (red) and vimentin (green) in a parallel slide from **c**, showing colocalization (yellow) in the cells with basal processes, suggesting that these cells are a subtype of glial radial cells that do not project to the cortical plate, instead restricted to the white matter tracts associated with gliogenic phenomena. Immunohistochemistry for AQP4 (**k**) and vimentin (**l**) in the subventricular zone shows expression in glial radial cells with apical and basal processes (arrow) and glial radial cells with single basal processes (arrowhead). M; Detail of basal projections of the SVZ indicating glial radial cell functionality as a scaffold to guide cells toward white matter tracts (nuclei labeled with DAPI in blue). Immunohistochemistry for AQP4 (**n**) and vimentin (**o**) in VZ showing expression in undifferentiated intermediate progenitor cell-like cells. P; Double labeling against vimentin and AQP4 at the VZ. Intermediate progenitor cell-like cells stain for vimentin only (arrowhead) and colocalizing with AQP4 (arrow). LV, lateral ventricle; VZ, ventricular zone; SVZ, Subventricular zone. Scale bars: **b** 500 µm; **c–j** 160 µm; **k, l** 30 µm; **m–p** 40 µm

At 21 weeks of gestation, AQP4 was expressed in association with central nervous tracts (CC and Fo), VZ, and SVZ (Fig. 3a–f). In these tracts, AQP4-positive cells had extensive basal processes that curved to follow the nerve fibers of these tracts (Fig. 3b, c, e). In the parietooccipital coronal section of a 21 PCW, CC fibers were observed to progress perpendicularly to the section plane, with fibers of the fornix progressing ventrally in the same section plane (Fig. 3b). AQP4 and vimentin colocalized on 100% of the cellular processes of fiber tracts and showed scaffold functionality guiding cells in the direction of the fiber tracts (Fig. 3m). However, AQP4 was not expressed in GRCs that project radially toward the cortical plate, as seen when labeled with vimentin (Fig. 3f, g). Thus, AQP4 selectively labeled GRCs that did not progress radially. Specifically, AQP4 was mainly expressed in basal GRC, but we also found expression that colocalized with vimentin in undifferentiated cells with the morphology of intermediate progenitors (Fig. 3k–p).

#### AQP4 and GFAP

**Fig. 4:**
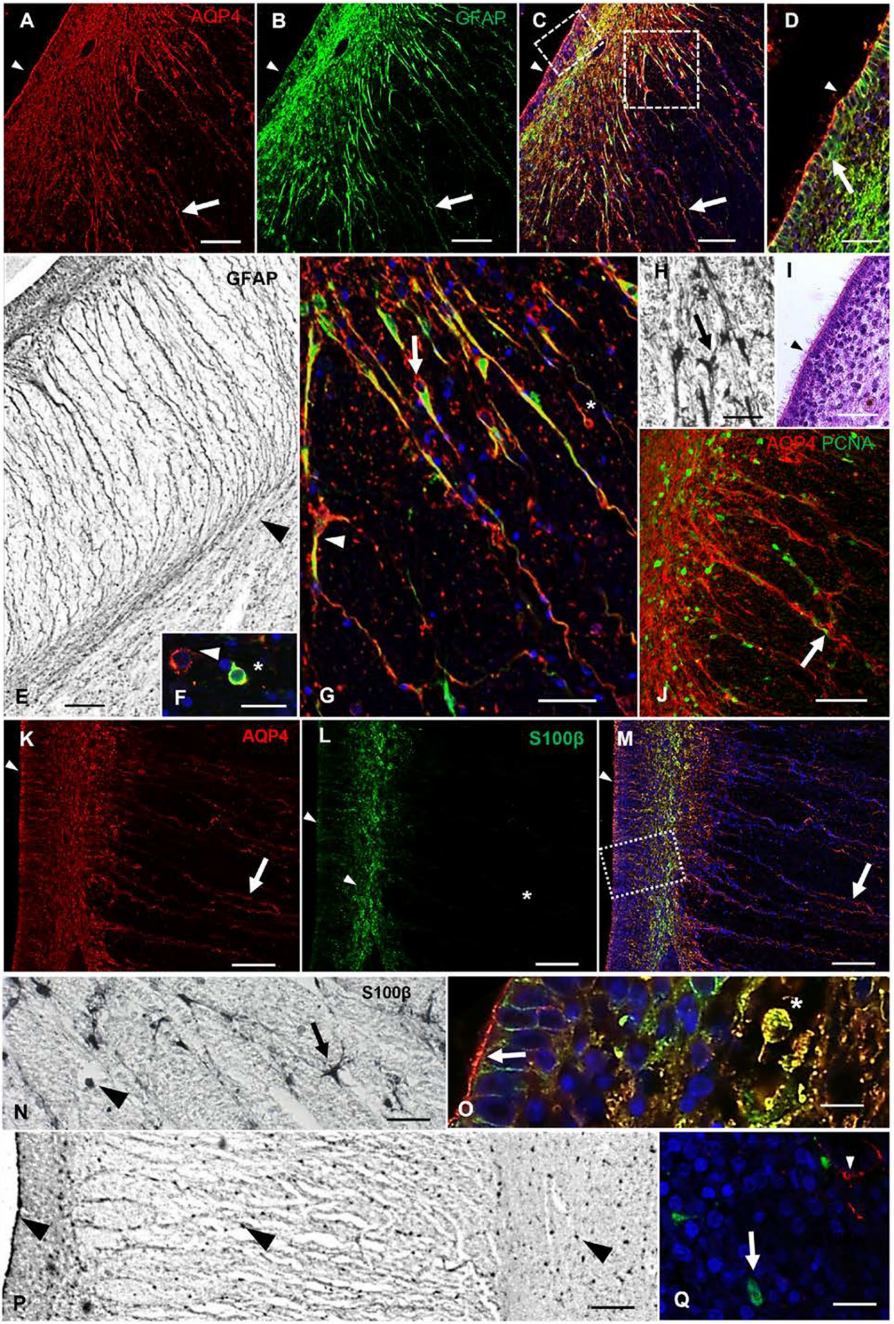
AQP4 and astrocytic markers in a control sample of 21 PCW. **a** AQP4 immunofluorescence. The arrow indicates immunodetection of AQP4 in cellular processes, while the arrowhead indicates immunodetection of AQP4 at the apical poles of ependymal cells. **b** Anti-GFAP immunofluorescence. Arrow indicates a positive GFAP radial process; arrowhead indicates the absence of GFAP at the apical pole of the ependyma. **c** Merge between **a** and **b**. Arrow indicates colocalization in cellular processes. Arrowhead indicates immunodetection of AQP4 at the apical pole of the ependyma. **d** Magnification of square in **c** showing VZ. Arrow points to positive GFAP immunostaining on basolateral walls, while AQP4 mostly labels the apical pole of the ependyma (arrowhead). **e** GFAP immunohistochemistry of the fornix region, showing GFAP staining in basal processes that curve to follow the fiber tracts of the fornix (arrowhead). **f** Detail of cells with intermediate progenitor morphology. Arrowhead points to AQP4 expression. Asterisks marks colocalization of AQP4 and GFAP. **g** Magnification of the subventricular zone in C. Arrow points to basal radial glial cells with AQP4 staining only in the cell body, but colocalized AQP4 and GFAP staining in the cell process. Arrowhead point to an AQP4-positive cell that is differentiating into an astrocyte. Asterisk marks a cell with basal radial glia morphology emitting a cellular process towards the apical zone. **h** Detail of the subventricular area near the fornix immunostained with anti-GFAP. Arrow points to an astrocyte. **i** Hematoxylin–eosin staining of a section stained in parallel with **d** Arrowhead points to characteristic multicilia on ependymal cells. **j** Immunolabeling against AQP4 and PCNA (mitosis marker) in a section stained in parallel with **g**. AQP4-positive cells are restricted to the white matter tracts and serve as a scaffold for proliferative cells. Arrow indicates a proliferative cell migrating through a radial process. **k** Anti-AQP4 immunofluorescence in a slide stained in parallel with **a**. Arrow indicates immunodetection of AQP4 in cellular processes. Arrowhead points to immunodetection of AQP4 at the apical pole of the neuroepithelium. **l** Anti-S100β immunofluorescence. Arrowheads point to the expression of S100β in the ependymal and subventricular cells. The asterisk indicates lack of S100β expression in the radial processes. **m** Merge between **k** and **l**. Arrow points to AQP4 staining in cellular processes. Arrowhead points to immunodetection of AQP4 at the apical pole of the ependyma. **n** Detail of S100ß expression in the subventricular zone. Arrow points to a differentiated astrocyte and arrowhead indicates an undifferentiated cell. **o** Magnification of the VZ in M. Arrow points to positive cytoplasmic immunostaining for S100β. Asterisk marks colocalization in the VZ. **p** Panoramic view of the medial parieto-occipital cortex labeled with anti-S100β, where the absence of radial processes is observed. Arrowheads indicate expression in cellular bodies of the ependymal cells and throughout the parenchyma. **q** Slide stained in parallel with P and double labeled with AQP4 (red) and S100β (green) at the cortical plate area, showing no colocalization between AQP4 (arrowhead) and S100ß (arrow). PCW, post-conceptional weeks; VZ, ventricular zone. Scale bar: **a, b, c, i** 200 µm; **d, g, h, n, q**, 40 µm; **e, p** 100 µm; **f, o** 20 µm; **j** 50 µm; **k–m** 80 µm

The expression of GFAP at 21 PCW was similar to that of vimentin, except that GFAP was also expressed by differentiated astrocytes (Fig. 4). Both AQP4 and GFAP marked intermediate progenitor-like cells and fiber GRC. Images obtained by confocal microscopy showed colocalization between AQP4 and GFAP in the cellular processes of the basal GRC of fiber tracts (Fig. 4a–c). Interestingly, these basal GRCs served as a scaffold to guide proliferative PCNA-positive cells toward the fiber tracts (Fig. 4j). In the VZ, AQP4 was expressed at the apical poles of ependymal cells, while GFAP was expressed in the basolateral membrane of the ependyma (Fig. 4d, i).

#### AQP4 and S100β

S100β expression was observed throughout the cortex. GRCs were not labeled with S100β. No colocalization was found in the IZ or white matter fiber tracts, indicating that cells expressing AQP4 belong to a different glial lineage than cells expressing S100β (Fig. 4k–q).

### AQP4 and hydrocephalus

**Fig. 5:**
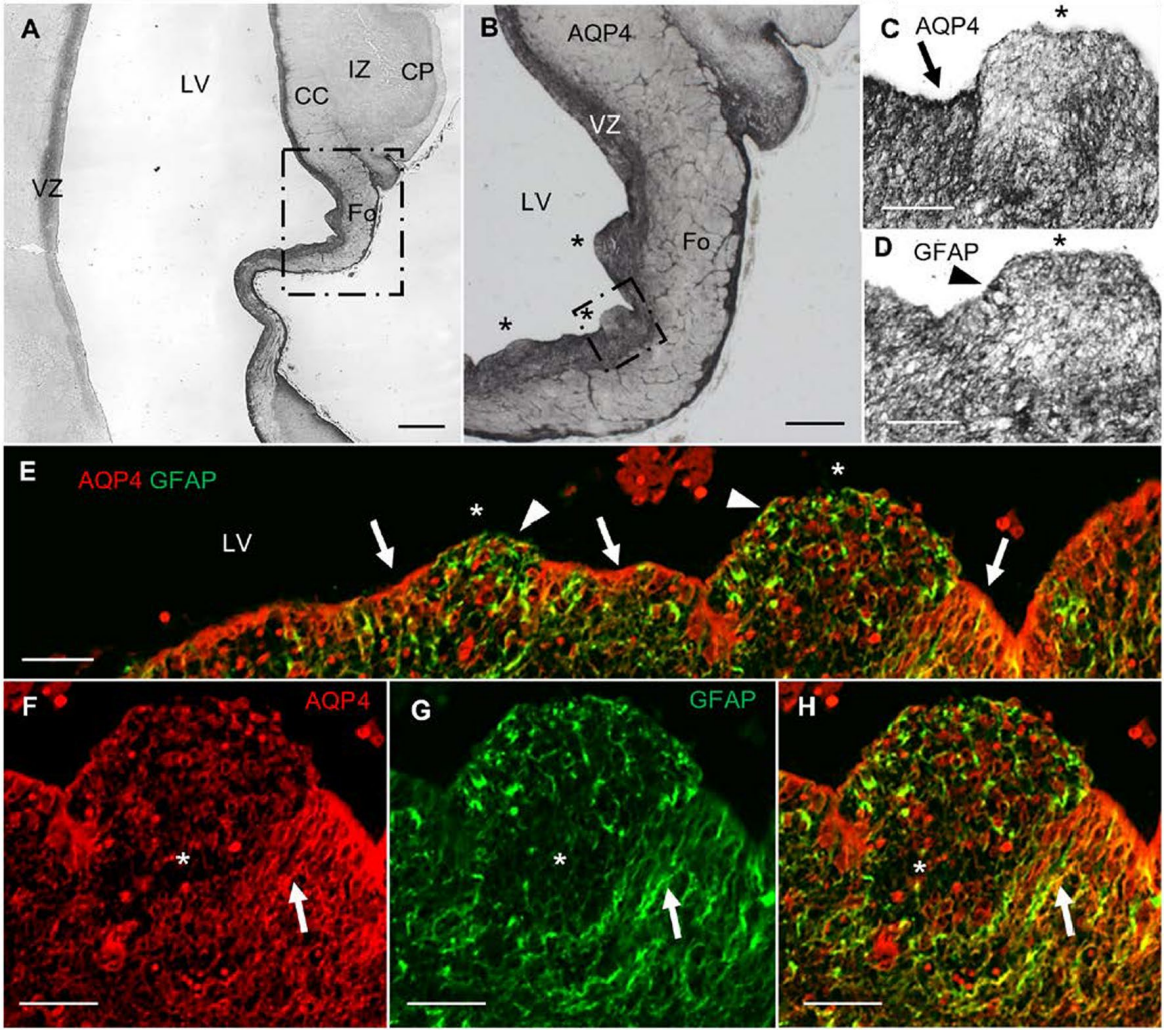
VZ disruption in a myelomeningocele case complicated with hydrocephalus. **a** Panoramic view of a coronal section of the parietooccipital area labeled with anti-AQP4. **b** Magnification of the square in **a**. Asterisks indicate areas of VZ disruption. **c** Magnification of the square in **b**. Arrow points to AQP4 expression lining the ventricle. Asterisk marks the area of disruption, showing characteristic protrusion toward the ventricle, without AQP4 expression lining the ventricle. **d** Slide stained in parallel with **c** for GFAP. Asterisk marks VZ disruption. Arrowhead marks cellular expression of GFAP. **e** Confocal microscopy of a slide stained in parallel with **b** for AQP4 (red) and GFAP (green). AQP4 is expressed along the lining of the ventricle (arrows) but not in the areas of VZ disruption (asterisk), where GFAP expression is dominant (arrowheads). **f–h** Detail of the VZ showing that AQP4-positive glial radial cells (arrows) are not present in areas of VZ disruption. CC, corpus callosum; CP, cortical plate; Fo, fornix; IZ, intermediate zone; LV, lateral ventricle; VZ, ventricular zone. Scale bar: **a** 500 µm; **b** 160 µm; **c–h** = 80 µm

**Fig. 6:**
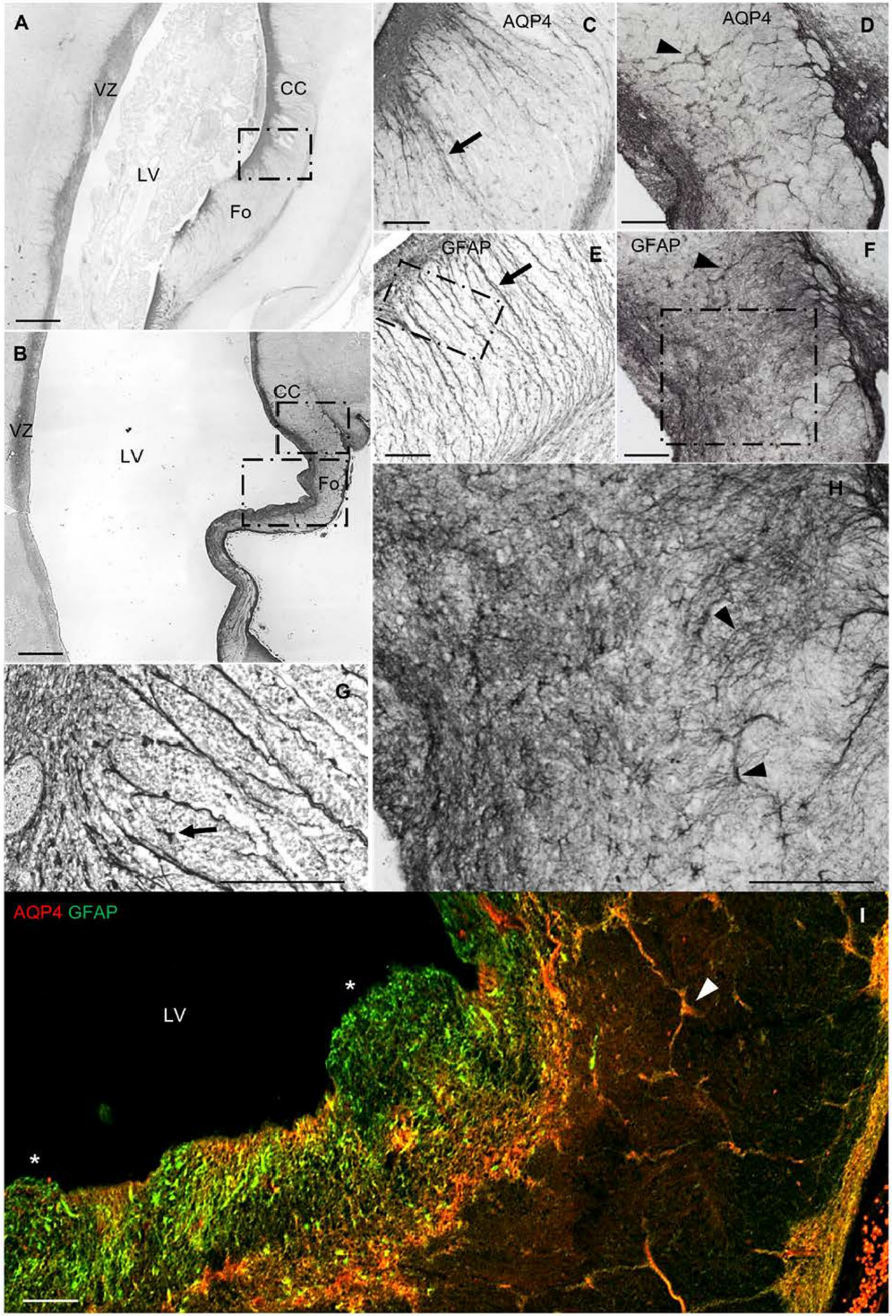
Expression of AQP4 and GFAP in control vs. hydrocephalic fetuses. **a** Panoramic image of a coronal section of a control brain at 21 PCW. **b** Panoramic image of a coronal section (same region as in **a**) from a brain with myelomeningocele at 21 PCW. **c** Magnification of the square in **a**, showing the ventricular area adjacent to the fornix and the corpus callosum in a control case. **d** Magnification of the ventricular area adjacent to the fornix and CC in a case with myelomeningocele complicated with hydrocephalus. **e** Slide stained in parallel with **c** for GFAP. **f** Slide stained in parallel with **d** for GFAP. Arrows point to glial radial cells. Arrowheads point to disorganized glial radial cells. **g** Detail of the control case with anti-GFAP. Arrow points to an undifferentiated GFAP-positive cell. **h** Image of the same region as in **g** from a patient with myelomeningocele complicated with hydrocephalus, immunostained with anti-GFAP. Arrowhead points to a differentiated astrocyte showing signs of reactive astrogliosis. **i** Slide stained in parallel with **b**, double-labeled with anti-GFAP and anti-AQP4. Asterisks mark VZ disruption. Arrowheads point to disorganized glial radial cells. CC, corpus callosum; Fo, fornix; LV, lateral ventricle; VZ, ventricular zone. Scale bar: **a, b** 500 µm; **c–h** 140 µm; **i** 80 µm

In the hydrocephalic subject, we found VZ disruptions adjacent to the areas of the CC and Fo. AQP4 apical expression was interrupted, no longer forming a line in the VZ (Fig. 5). These disruptions were accompanied by SVZ protrusions into the ventricular cavity. The expression of GFAP was dominant in disrupted areas, indicating that reactive astrocytes had covered the disrupted area. There was a lack of GRCs associated with the VZ disruption (Fig. 5f–h). In the periventricular white matter of the SVZ, we found reactive astrogliosis proximal to the disrupted areas (Fig. 6). Activated glia expressed both GFAP and AQP4. The expression of GRC was disorganized under hydrocephalic conditions, suggesting that these cells were differentiating into astrocytes as a response to the pathology.

### Expression of AQP4 and GFAP in microvesicular fractions

The results of western blotting showed AQP4 expression in "pellet" 2 and "pellet" 3, which corresponded to the vesicular fractions of CSF. However, AQP4 expression was not present in the total protein fraction from S3, which indicates that the expression of AQP4 is associated with vesicles in the CSF.

**Fig. 7:**
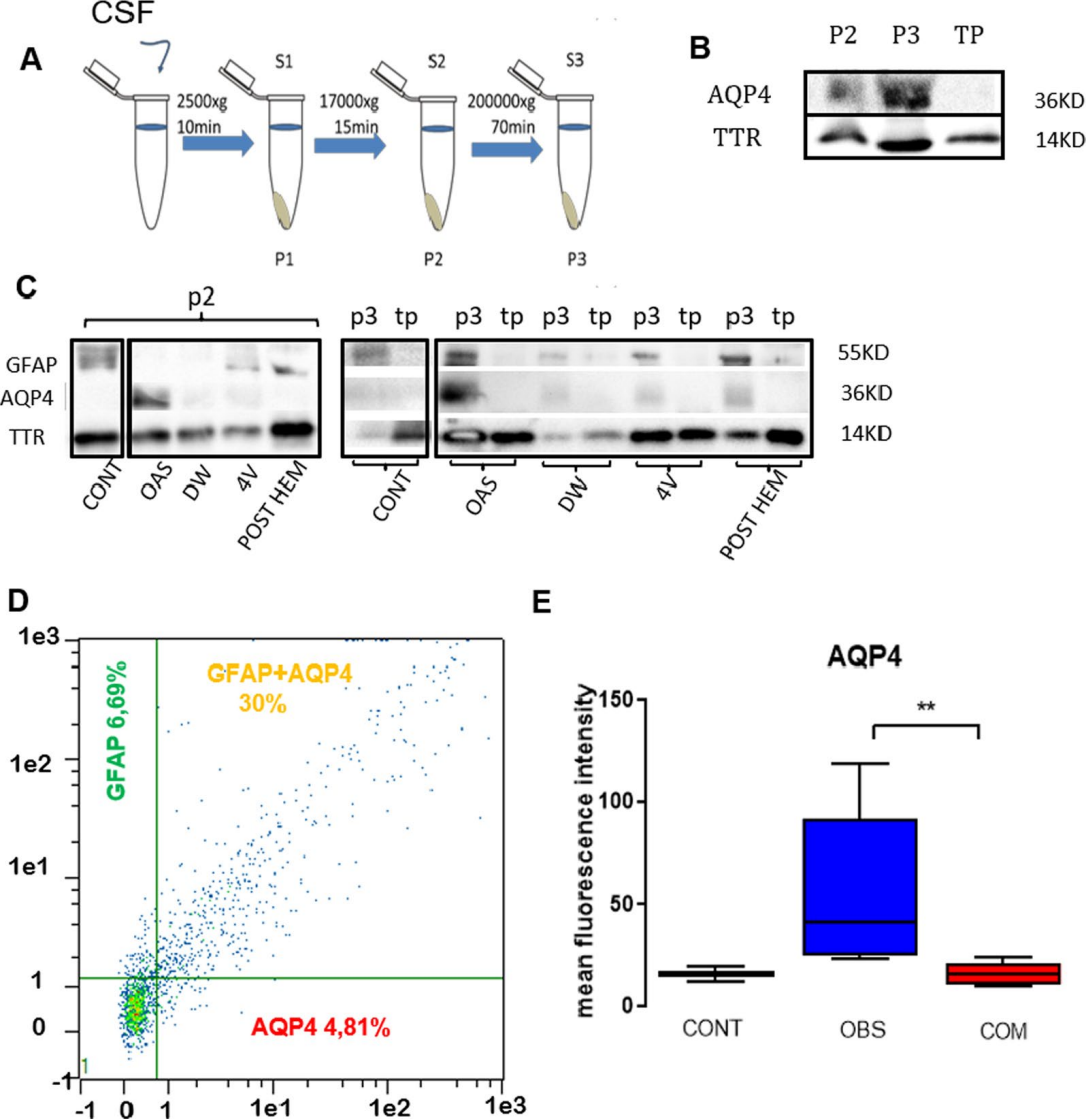
Expression of AQP4 and GFAP in the micro vesicular fraction of the CSF. **a** Schematic representation of vesicular extraction. **b** Expression of AQP4 is specifically found in the P2 and P3 fractions and is absent in the total protein fraction. TTR (14KD) was used as a load control since its expression was found in all fractions, including total protein obtained from supernatant 3. **c** Expression of GFAP and AQP4 in vesicular fractions from pellet 2 and pellet 3. No expression was found in total protein from supernatant 3. **d** Graphic representation of the colocalization between AQP4 and GFAP, with few events that present only AQP4 or only GFAP. **e** Graphic representation of fluorescence intensity showing increased AQP4 expression in the microvesicular fraction of CSF from patients with obstructive hydrocephalus. CSF, cerebrospinal fluid. S, supernatant; P, pellet; TP, total protein; 4V, tetraventricular hydrocephalus; CONT, control; DW, Dandy Walker malformation; OAS, mesencephalic aqueduct obstruction, POST HEM, post-hemorrhagic hydrocephalus; OBS, obstructive hydrocephalus; COM, communicating hydrocephalus.

Similarly, GFAP expression was also linked to vesicular fractions, with no expression observed in the free protein fraction from S3 (Fig. 7b, c). AQP4 expression was not detected in the microvesicular fraction of control samples.

### Flow cytometry

Increased AQP4 microvesicle expression was confirmed with flow cytometry in which the mean fluorescence intensity of AQP4 was significantly higher in samples from patients with obstructive hydrocephalus, (41.13; *p* < 0.01) compared to samples from patients with communicating hydrocephalus (15.87; *p* < 0.01) or control (15.86; *p* > 0.05). Also, GFAP showed not significantly higher levels of expression in obstructive cases (control, 137.2; obstructive, 243.9; communicating, 175.3; *p* > 0.05). The percentage of colocalization between GFAP and AQP4 showed no differences (control, 32,29; obstructive, 37.68; communicating, 31.84; *p* > 0.05) (Table 4, Fig. 7d, e).

**Table 4:** Flow cytometry data

Sample	GFAP (MFI)	AQP4 (MFI)	Colocalization (%)	Classification
1	297.42	49.11	32.2	Obs
2	29.58	22.96	11.05	Obs
3	1008.82	118.7	87.81	Obs
4	45.27	33.15	11.6	Obs
5	344.21	82.06	57.58	Obs
6	190.02	25.66	43.16	Obs
Obs median	243.9	41.13	37.68	
7	126.76	20.12	28.35	Com
8	283.12	15	56.08	Com
9	77.59	10.83	35.32	Com
10	43.25	10.01	16.78	Com
11	286.9	23.86	46.22	Com
12	223.76	16.73	26.2	Com
Com median	175.3	15.87	31.84	
13	226.55	19.66	45.44	Cont
14	47.94	12.05	19.14	Cont
Cont median	137.2	15.86	32.29	Cont

*Com* communicating hydrocephalus, *Cont* control, *Obs* obstructive hydrocephalus, *MFI* mean fluorescence intensity

The samples that were processed for the flow cytometer were also observed by confocal microscopy to confirm the presence of different pools of microvesicles: < 1 µm, 1–2 µm, and 2–4 µm (Additional file 1: Fig. S2).

## Discussion

### AQP4 expression throughout the developing brain

With the discovery of AQP4, whose expression is mainly localized in astrocyte end-feet, researchers began to understand the molecular basis of water transport across cortical parenchymal membranes. This aqueous channel is linked to the balance of central nervous system fluids, with increased expression contributing to the resolution of brain edema or serving as a compensatory mechanism for hydrocephalus [6, 10]. Astrocytes have been reported to participate in synaptogenesis, the maintenance of synaptic activity, and nerve transmission through the interstitial fluid for homeostasis. Thus, astrocyte end-feet surround the synapses and nodes of Ranvier to maintain adequate levels of pH, ions, neurotransmitters, and fluid [3, 66]. Specifically, AQP4 modulates aqueous flow for clearance of the K + produced during nerve transmission [3, 66]. The presence of excess protons maintains a hyperpolarized state that would prevent the passage of a new nerve impulse. Similarly, in the glymphatic system, AQP4 plays a key role in modulating the aqueous flow to clear parenchymal waste released during brain activity [62]. Failed clearance of the parenchymal interstitium is associated with normal-pressure hydrocephalus [56, 59], Alzheimer disease [32], traumatic brain injury [31], stroke [25], and neonatal hydrocephalus [48]. In this study, samples were obtained from brains that ranged in age from 8 PCW to adulthood to thoroughly study human AQP4 expression.

We propose that the expression of AQP4 in human fetal development may be associated with glymphatic system activity and parenchymal fluid flow. Few studies have investigated the expression of AQP4 during human fetal development. Gömöri et al. [28] reported expression of AQP4 from 14 to 40 PCW, and Bjornbak Holst et al. [5] reported expression from 12 to 21 PCW.Based on our results, AQP4 begins to be expressed at 13 PCW in cell bodies and cellular projections of the glioepithelium of the fimbria of the dorsal hippocampus (Fig. 1), which is consistent with previous findings [5]. AQP4 expression progresses toward significant fibrous tracts, such as the CC, the fornix, and the internal capsule in medial areas of the brain. AQP4 is also expressed in the LGE (Fig. 2). From 25 PCW onward, AQP4 is expressed throughout the neocortex, mainly in the intermediate zone or subplate, an area that will later give rise to the white matter of the cerebral cortex. At precisely 25 PCW, AQP4 expression was seen for the first time in the subplate, in astrocyte end-feet surrounding the blood vessels and forming the typical neurovascular unit in the neocortex (Fig. 2). Therefore, AQP4-dependent parenchymal flow (from the perivascular space to the interstitium and inversely) in the glymphatic system is only possible after this time in development. Before that, AQP4 is mainly expressed in the main fibrous tracts of the Isocortex, possibly to provide homeostasis to facilitate neural impulse transmission to incipient fiber tracts. In general terms, AQP4 expression progresses from medial to lateral, starting in the archicortex of the dorsal and ventral hippocampus, followed by the ganglionic eminences, and continuing with the neocortex: initially at the perisylvian regions, and lastly in the occipital and prefrontal zones, as reported by Bjornbak Holst et al. [5]. We suggest that the expression pattern of AQP4 could define a developmental program for cortical nervous functionality, with archicortex (primitive cortex) acquiring functionality early during gestation, while the frontal and occipital poles acquire functionality later in gestation. We posit a functional and evolutive logic, with early maturation in the most primitive telencephalic cortex and late maturation in the telencephalic poles responsible for vision and cognitively complex behaviors, which are unnecessary in utero.

### AQP4 expression throughout gliogenesis

Classically, astrocytes were considered a homogeneous population of nerve cells. However, tremendous morphological and functional diversity was confirmed, with astrocytes playing fundamental roles during synaptogenesis in cortical development [64]. Several disorders, including pediatric hydrocephalus [60] and Rett syndrome [42], are associated with impaired glial activity during central nervous system development. Therefore, a precise understanding of glial development is crucial to understanding astrocytes' functional and anatomical heterogeneity. It has been suggested that astrogenesis progresses through at least four stages: a proliferative radial glia phase, a stage of intermediate progenitors (glioblasts), another of immature astrocytes, and finally the adult astrocyte phase [1, 47]. However, defining the role of astrocytes in neurological disorders is complicated due to the absence of biomarkers that allow us to distinguish between potentially different astrocyte subtypes and how they evolve during cortical development [5, 24, 46, 47]. AQP4 is an astrocytic glial marker that has scarcely been studied in the different stages of glial genesis. According to our results, AQP4 is a marker of the entire process of astrocytogenesis since, throughout brain development, AQP4 labels GRCs, cells with intermediate progenitor morphology, and astrocytes. Interestingly, we found that the expression of AQP4 was associated with fibrous tracts. Even in adults, we found that AQP4 mainly labeled the entire soma and processes of fibrous astrocytes in white matter, while in grey matter, we found AQP4 mainly expressed in areas surrounding vessels.

#### AQP4 is expressed in glial radial cells

Our results show that a specific AQP4-positive subgroup of GRCs is associated with crucial white matter tracts and transient cortical structures, such as the ganglionic eminences. At approximately the 25th week of gestation, AQP4-positive glia was observed throughout the cortex, mainly in the subventricular and intermediate zone, giving rise to white matter after neurogenesis had ended. This temporospatial progression of AQP4 expression was associated with radial glial cells that will specifically give rise to astrocytes. Firstly, AQP4 was detected in the archicortex, which is associated with more primitive phylogenic maturation structures during early development. Next, AQP4 is expressed in the ganglionic eminences, structures that contribute to development (first neurogenic, later gliogenic) and ultimately degenerate. Finally, AQP4 is observed in the rest of the neocortex, mainly associated with white matter. To confirm that AQP4 specifically marks radial glia, we performed double-labeling studies with confocal microscopy, using vimentin as a nonspecific GRC marker [67]. As shown in the results, both proteins colocalized, with AQP4 labeling apical GRCs (attached to the VZ) and basal GRCs (not attached to the VZ), which confirms the usefulness of AQP4 as a marker for GRCs (Fig. 3).

On the other hand, the astrocyte marker used most commonly is GFAP [47]. However, at prenatal ages, GFAP marks both differentiated astrocytes and GRC. AQP4 colocalizes very specifically with GFAP, in GRC linked to the main fibrous tracts, which suggests that this aqueous channel is specifically marking a type of GRCs that will give rise to astrocytes (Fig. 3). When we double-labeled AQP4-positive cells with S100ß, a widely used astrocytic marker, we found that the AQP4-positive GRC did not express S100ß, and no colocalization was found at the cortical plate levels, which indicates that S100ß and AQP4 may label different astrocytic lineages such as fiber astrocytes or protoastrocytes. Vimentin and GFAP label all GRCs throughout the cortex at mid-gestation, while S100ß does not label GRCs. Therefore, AQP4 seems to be a gliogenesis marker since it selectively labels GRC associated with the white matter, where neurogenesis has ended and gliogenesis continues. We propose that AQP4 reflects developmental glial contribution to the cortex.

Interestingly, these AQP4-positive GRCs do not have a classical radial morphology since they do not progress radially from the ventricular zone. However, they curve along central white matter tract fibers, serving as a scaffold for proliferative cells migrating toward these white matter areas. Therefore, due to its glial lineage and GRC functionality, we believe that the appropriate name for these cells would be glial stem cells (GSC).

#### AQP4 is expressed in intermediate progenitor cells (glioblasts)

In addition to labeling the cell population coined for the first time in this manuscript as GSC, AQP4 also marks undifferentiated cells with intermediate progenitor morphology. At the VZ, AQP4 cells with the morphology of intermediate progenitors stain for vimentin and GFAP (Figs. 3, 4), suggesting that these cells are glioblasts. Therefore, AQP4 seems to label different stages of gliogenesis differentiation, including GSCs with basal processes that follow the central white matter fiber tracts, glioblasts, and astrocytes.

### AQP4 in hydrocephalus

Hydrocephalus is a nervous system disorder mainly characterized by impairment in the CSF flow, ventriculomegaly, VZ disruption, reactive astrogliosis, disordered neurogenesis, and CC malformations [20, 29, 57]. AQP4 is the main water channel present in the central nervous system, located mainly in the end-feet of perivascular astrocytes, at the CSF-brain parenchyma interface, either in the ependymal membranes or in the end-feet of subpial astrocytes and perisynaptic areas [2]. AQP4 is mainly associated with the reabsorption of cerebral edema and parenchymal flow. Several systematic studies have focused on the implications of water channels in the pathophysiology of pediatric hydrocephalus, concluding that there is a change in AQP protein expression, as a compensatory mechanism for hydrocephalus, to increase the reabsorption and decrease the production of CSF [6, 10, 14, 23, 60, 62]. AQP4 is also linked to the stability and maturation of the ependyma [40]. In fact, it has been shown that 40% of AQP4 knockout mice have VZ disruption, with 10% also showing hydrocephalus due to stenosis of the Sylvian aqueduct [22]. Therefore, AQP4 functionality seems not to be restricted to modulation of the parenchymal fluid. Our analysis of samples from a patient with hydrocephalus showed VZ disruption associated with protrusion of the SVZ into ventricular space, which is characteristic of pediatric hydrocephalus [20, 29, 44]. In the disrupted areas of the VZ, the lining of AQP4 expression was discontinuous due to a loss of ependymal cells. However, we found expression of GFAP-positive cells throughout the protrusions in disrupted areas. Interestingly, we found a lack of "GSCs" in the disrupted areas. This cellular loss could explain the characteristic CC dysgenesis associated with hydrocephalus, since proliferative cells migrate toward the central white matter fiber tracts using these GSC (Fig. 5).

#### Reactive astrogliosis

Reactive astrogliosis is characterized by hyperplasia and hypertrophy of the astrocytes. Astrocytic reactions have been shown to inhibit axonal regeneration [9, 21]. However, reactive astrogliosis has also been associated with the release of growth factors and trophic molecules that promote neuronal growth such as nerve growth factor (NGF) or basic fibroblast growth factor (bFGF) [58]. Therefore, the benefits and consequences of glial activation are highly controversial. Some authors consider reactive astrogliosis as a cause of damage during cortical development. Although the use of anti-inflammatory compounds has been effective in treating hydrocephalus in animal models [8, 43] some authors maintain that the astrocyte reaction protects brain tissue and contributes to its functional recovery [27]. Roales-Buján et al. [60] found that glial activity repairs the VZ by expressing AQP4 and replacing disrupted VZ. Our results showed GFAP-positive cells in disrupted areas, possibly as replacements for ependymal cells. We detected reactive astrogliosis adjacent to the disrupted areas, close to CC and Fo. These activated cells expressed AQP4 and GFAP and were associated with disorganized GSC. In contrast, in control cases, GSCs expressing AQP4 and GFAP remained undifferentiated. This finding could indicate early differentiation of GSC and glioblasts into reactive astrocytes. Thus, pathological conditions associated with pediatric hydrocephalus could involve a change in cell fate, with cells previously committed to supporting cellular proliferation in white matter differentiating into reactive astrocytes. This gliogenic alteration would explain the characteristic CC dysgenesis associated with pediatric hydrocephalus [53] (Fig. 7). Since our findings are based on qualitative histology changes, further studies need to be done for quantitative confirmation.

### AQP4 is expressed in microvesicles as a possible hydrocephalus biomarker

An increase in the production of microvesicles in the CSF has been associated with reactive astrogliosis [65]. Since glial activation is a characteristic phenomenon in hydrocephalus, where AQP4 is expressed in astrocytes [60], we propose that the presence of AQP4 in the CSF of pediatric hydrocephalic patients could derive from microvesicles released by astrocytes. We isolated microvesicles and tested for the presence of GFAP and AQP4. Our results showed that GFAP and AQP4 were present in the vesicular fractions (P2 and P3) but not in the fraction with total protein from S3. Therefore, we suggest that both proteins are part of vesicles and are not free in the CSF (Fig. 7). AQP4 was not detected in control CSF samples, which indicates potential for the use of AQP4 as a biomarker. Using flow cytometry, we demonstrated that the GFAP-positive vesicles produced by glial cells colocalized mostly with AQP4 and that AQP4 levels were higher in samples from patients with obstructive hydrocephalus (Fig. 7). Future studies that include the quantification of AQP4 in CSF could provide information about disease status, allowing for improved prognosis as well as the design of appropriate treatments for specific cases of hydrocephalus.

## Conclusion

AQP4 is expressed in the end-feet of astrocytes forming the neurovascular unit after 25 PCW under normal conditions. Neuropathological effects associated with hydrocephalus such as reactive astrogliosis and glial stem cell disorganization are observed before 25 PCW. In addition, microvesicles from CSF in patients with obstructive hydrocephalus show increased AQP4 expression.

## Supplementary Information

**Additional file 1: Figure S1**. Expression of AQP4 in control samples, complementary results. A; No AQP4 expression was detected at 8 PCW. B; No AQP4 expression is detected at 10 PCW. C; No expression is detected in the occipital area at 13 PCW, indicating a delay of the expression of AQP4 (asterisk). D & E; Expression at 15 PCW remained associated with the archicortex (arrows). F; AQP4 expression is present throughout the parietooccipital section of a 25 PCW brain. G; Image of a coronal section of white matter from parieto-occipital neocortex in a 34-year-old brain. H; Magnification of the square in G. Arrowhead points to an astrocyte. I; Image of gray matter in parieto-occipital neocortex showing that AQP4 expression is mostly restricted to the neurovascular unit (arrowhead). DHip, dorsal hippocampus; Fo, fornix; GE, ganglionic eminence; Htha, hypothalamus; LV, lateral ventricle; PCW, post-conceptional weeks; PM, pia mater; Tha, thalamus; VC, Visual cortex; VHip, ventral hippocampus; VZ, ventricular zone. Scale bars: A & B = 800 µm; C, 300 µm; D & F, 600 µm; E, 70 µm; G, 80 µm; H & I: 40 µm. **Figure S2**. Representative images of microvesicles present in CSF samples. CSF, cerebrospinal fluid. Scale bar, 5 μm.

## Data Availability

The datasets during and/or analysed during the current study available from the corresponding author on reasonable request.
