# Supplementary figures and images for: AQP4 labels a subpopulation of white matter-dependent glial radial cells affected by pediatric hydrocephalus, and its expression increased in glial microvesicles released to the cerebrospinal fluid in obstructive hydrocephalus
Source: Acta Neuropathol Commun. 2022 Mar 28;10:41. doi: 10.1186/s40478-022-01345-4 (PMC8962176; doi:10.1186/s40478-022-01345-4)

Supplemental Figure 1.


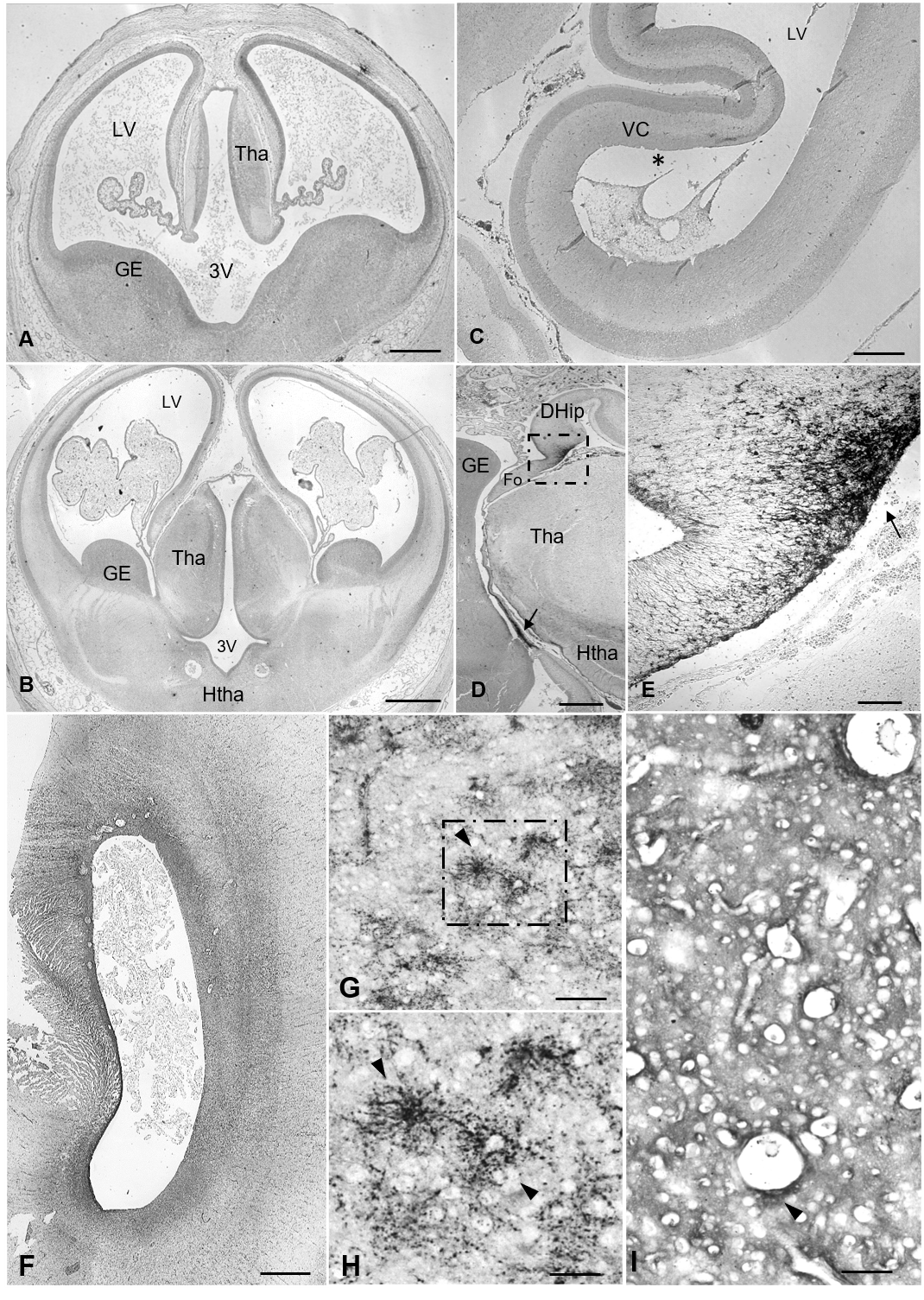


Supplemental Figure 2.


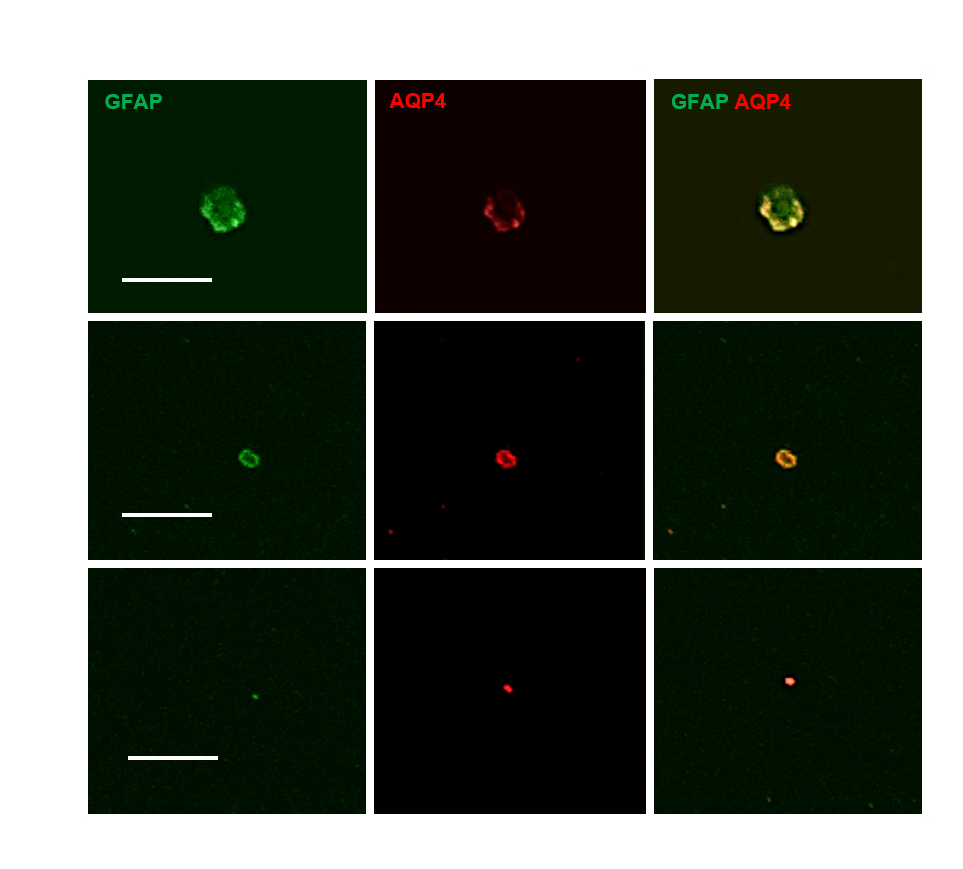

Supplement: Supplementary file 1 — Additional file 1: Figure S1. Expression of AQP4 in control samples, complementary results. A; No AQP4 expression was detected at 8 PCW. B; No AQP4 expression is detected at 10 PCW. C; No expression is detected in the occipital area at 13 PCW, indicating a delay of the expression of AQP4 (asterisk). D & E; Expression at 15 PCW remained associated with the archicortex (arrows). F; AQP4 expression is present throughout the parietooccipital section of a 25 PCW brain. G; Image of a coronal section of white matter from parieto-occipital neocortex in a 34-year-old brain. H; Magnification of the square in G. Arrowhead points to an astrocyte. I; Image of gray matter in parieto-occipital neocortex showing that AQP4 expression is mostly restricted to the neurovascular unit (arrowhead). DHip, dorsal hippocampus; Fo, fornix; GE, ganglionic eminence; Htha, hypothalamus; LV, lateral ventricle; PCW, post-conceptional weeks; PM, pia mater; Tha, thalamus; VC, Visual cortex; VHip, ventral hippocampus; VZ, ventricular zone. Scale bars: A & B = 800 µm; C, 300 µm; D & F, 600 µm; E, 70 µm; G, 80 µm; H & I: 40 µm. Figure S2. Representative images of microvesicles present in CSF samples. CSF, cerebrospinal fluid. Scale bar, 5 μm. [file 40478_2022_1345_MOESM1_ESM.docx]
